# csaw: a Bioconductor package for differential binding analysis of ChIP-seq data using sliding windows

**DOI:** 10.1093/nar/gkv1191

**Published:** 2015-11-17

**Authors:** Aaron T.L. Lun, Gordon K. Smyth

**Affiliations:** 1 The Walter and Eliza Hall Institute of Medical Research, 1G Royal Parade, Parkville, VIC 3052, Australia; 2 Department of Medical Biology, The University of Melbourne, Parkville, VIC 3010, Australia; 3 Department of Mathematics and Statistics, The University of Melbourne, Parkville, VIC 3010, Australia

## Abstract

Chromatin immunoprecipitation with massively parallel sequencing (ChIP-seq) is widely used to identify binding sites for a target protein in the genome. An important scientific application is to identify changes in protein binding between different treatment conditions, i.e. to detect differential binding. This can reveal potential mechanisms through which changes in binding may contribute to the treatment effect. The csaw package provides a framework for the *de novo* detection of differentially bound genomic regions. It uses a window-based strategy to summarize read counts across the genome. It exploits existing statistical software to test for significant differences in each window. Finally, it clusters windows into regions for output and controls the false discovery rate properly over all detected regions. The csaw package can handle arbitrarily complex experimental designs involving biological replicates. It can be applied to both transcription factor and histone mark datasets, and, more generally, to any type of sequencing data measuring genomic coverage. csaw performs favorably against existing methods for *de novo* DB analyses on both simulated and real data. csaw is implemented as a R software package and is freely available from the open-source Bioconductor project.

## INTRODUCTION

The ChIP-seq technique identifies protein–DNA interactions by massively parallel sequencing of DNA bound to a target protein. ChIP-seq is often used to find the binding sites of a transcription factor (TF) or to examine the positioning of a histone mark across the genome. It is a key tool for investigating the function of DNA-binding proteins, for identifying novel DNA elements, and for studying the molecular mechanisms of gene regulation. Traditional analyses of ChIP-seq data involve identifying peaks of high read density in the genome, using software like MACS (1), HOMER (2) or SICER (3). These peaks represent putative binding sites for the target protein. Binding sites are then considered present or absent in each sample, allowing qualitative comparisons between DNA samples or experimental conditions. An alternative strategy that is beginning to receive more attention is to identify quantitative changes in the binding profile between experimental conditions, i.e. to analyze differential binding (DB) (4–7). The DB approach allows a more rigorous statistical analysis to be formulated. It also focuses directly on sites that are associated with biological differences between the samples and hence may have biological significance. By contrast, strongly bound sites detected by peak calling may not necessarily be biologically interesting if the intensity of binding does not change between treatment conditions.

One can discriminate between DB analyses for which the genomic intervals over which DB is tested are specified in advance and *de novo* analyses where the intervals are *a priori* unknown. Pal *et al*. (5) conducted a gene-oriented analysis of DB, whereby they tested for DB between cell populations at genomic intervals defined by the transcriptional start and stop positions of each gene. This type of DB analysis can be performed using essentially the same statistical methods as those used in a gene-based differential expression analysis of RNA-seq data (8,9). In many or most cases, however, it is of interest to find differentially bound regions anywhere in the genome without making any prior assumptions about the locations of the binding sites. For such *de novo* DB analyses, statistically rigorous assessment of DB is more subtle. This is because the genomic intervals over which DB is tested have to be empirically determined from the same data that is used to conduct those tests.

The earliest approach for *de novo* detection of differentially bound (DB) regions has been to use MACS or HOMER to call peaks from the data, and to use these empirical peaks as the regions of interest. Read counts can be obtained for each peak in each library, and analyzed with software like edgeR (10) to identify significant DB between conditions. This peak-based strategy is implemented in the Bioconductor software packages DiffBind (4) and DBChIP (11). Despite its popularity, this strategy has some potential problems that are not immediately obvious. We have shown previously that calling peaks in individual libraries or treatment groups can lead to loss of error rate control during the DB analysis (12). This is because the definition of the regions to be used for DB testing is not independent of the DB status of those regions. Moreover, imprecise calling of peak boundaries can decrease power to detect DB for sharp features such as TF binding sites (12). Power can also be lost for complex DB events involving changes in the shape of the binding profile. Such events are not uncommon for protein targets with broad enrichment, e.g. when histone marks shift or spread between conditions. Defining the entire site as a single peak will only consider overall changes in binding across the site, and may not capture DB in a specific subinterval of that site.

To avoid the biases and loss of resolution associated with peak calling, the software packages USeq (13), diffReps (14) and PePr (15) have implemented windowing strategies. Windows of constant size are placed at regular intervals across the genome, and each window is tested for DB. In this manner, *de novo* detection can be performed while avoiding peak calling altogether. This avoids problems with loss of error control at the window level, as—unlike peaks—the windows are defined independently from the data. To correct for multiple testing, most of these packages attempt to control the false discovery rate (FDR) across windows. However, DB results are often interpreted at the level of aggregated regions formed by combining groups of neighboring windows, and none of the existing software packages are able to control the FDR when DB discoveries are reported in terms of regions. We have shown previously that simply consolidating adjacent DB windows into DB regions leads to a loss of FDR control, because a given FDR at the window level does not translate into the same FDR at the region level (12).

Here we present csaw, a new window-based software package for *de novo* DB analyses of ChIP-seq data as part of the Bioconductor project (16,17). A key aim of csaw is to provide statistically rigorous FDR control across the reported regions. csaw tests for DB at the window level using a quasi-likelihood approach that robustly accounts for biological variation between replicates (9,18). csaw then combines *P*-values from adjacent windows in a manner that maintains FDR control at the level of the consolidated regions reported to the user.

The csaw package is designed to be flexible and modular. It assumes a replicated ChIP-seq experiment with at least two experimental conditions, where multiple biological replicates are present in at least one of the conditions. Experimental designs of any complexity can be accommodated, whether they involve multiple treatment factors, quantitative covariates, paired samples or batch effects. Similarly, any contrast between the treatment conditions can be formulated and tested, including non-trivial ANOVA-like or interaction-based contrasts.

csaw can be used with any type of ChIP-seq data, regardless of the protein target or whether sequencing is single- or paired-end. csaw does not require genomic annotation. csaw provides a range of normalization options, some not available in any earlier software. It can be used with or without negative control libraries such as input or IgG controls.

This article provides a brief description of the functionality in the csaw package. Several simulations are performed to highlight the advantages of using csaw over existing methods for *de novo* DB detection. Comparisons are made with a popular peak-based method, DiffBind, and with the most recent of the window-based methods, PePr. csaw is shown to provide greater or comparable detection power while still maintaining FDR control. The ability of csaw to detect complex DB events is demonstrated with a case study on real H3K4me3 data. These results show that csaw is a viable alternative for DB analyses of ChIP-seq datasets.

## MATERIALS AND METHODS

### Simulated datasets

Simulations were conducted to demonstrate the performance of csaw and other software tools in a range of situations. Separate simulations were conducted to represent the type of binding events typical of histone marks and TFs respectively. In the latter case, relatively sharp peaks were simulated. In the former case, relatively broad peaks were simulated, with special attention given to complex DB events in which the bound region may be extended or altered between conditions. Here we describe the design details of each simulation setup. Complete code necessary to reproduce these simulations can be downloaded from http://bioinf.wehi.edu.au/csaw.

#### Complex DB events

**Figure 1.**
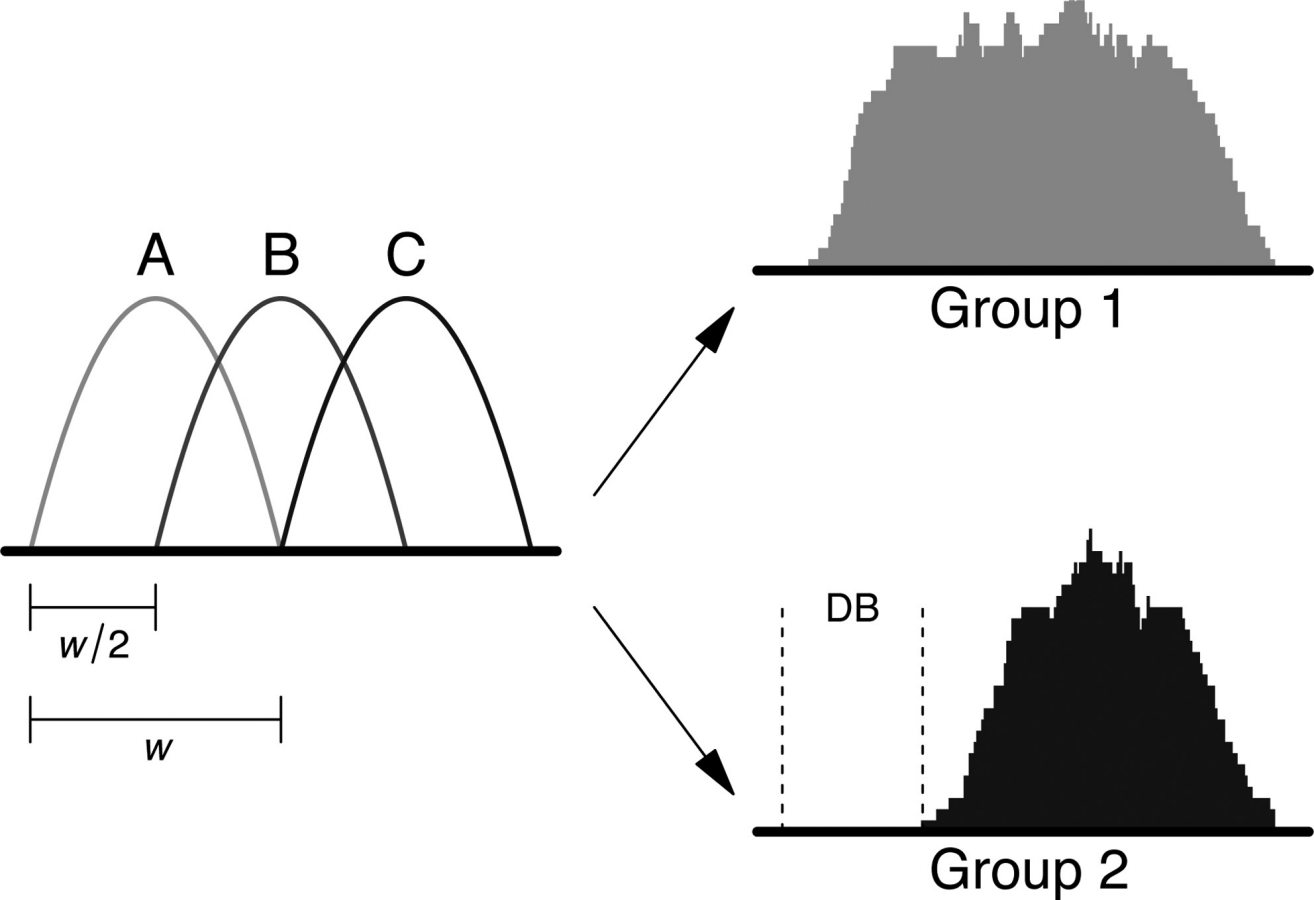
Diagram of the simulation design for complex DB events. The binding profile is comprised of subintervals A, B and C, each of width $w$ and offset from each other by $w$/2. DB is introduced by dropping one or two subintervals in one of the groups, e.g. subinterval A is dropped in group 2, above.

A histone mark ChIP-seq experiment was simulated with two biological replicates in each of two groups. A total of 20 000 binding sites spaced 10–20 kbp apart were simulated on one chromosome. To construct a complex event, the binding profile for each site was characterized as a mixture of three subintervals (Figure 1). Each subinterval was parameterized as a scaled Beta(2, 2) distribution with width $w$ = 500 bp. A Beta distribution was chosen to generate a smooth binding profile, consistent with real data. For each subinterval at site *i*, the number of reads in each library was sampled from a negative binomial (NB) distribution with a mean of 30 and a dispersion of ϕ_*i*_. This choice of mean corresponds to a binding site of moderate intensity, such that sufficient counts are present for statistical analysis. The value of ϕ_*i*_ was sampled from an inverse chi-squared distribution on 20° of freedom. This introduces variability in the dispersions across different sites, which is more realistic than having a constant dispersion for all sites. Read positions were sampled from *wX* + *s*_*j*_, where *X* ∼ Beta(2, 2) and *s*_*j*_ is the start location of subinterval *j*. Reads were randomly distributed between strands, as strand bimodality is less pronounced in histone mark data.

To introduce DB at a binding site, one or two subintervals were randomly chosen from that site. All reads associated with the chosen subintervals were removed from all libraries in one of the groups. This yields a complex DB event involving a change in the shape of the binding profile (Figure 1). This was performed for 500 sites in one group, and repeated for a different set of 500 sites in the other group. Balanced removal of reads ensures that composition bias is not introduced, avoiding the need for any normalization.

Non-specific background enrichment was also added to the simulation. This was performed by partitioning the genome into 2 kbp bins. For each library, the number of reads in each bin *k* was sampled from a NB(μ_*k*_, ϕ_*k*_) distribution. The mean μ_*k*_ was sampled from a uniform distribution on the interval (10, 50) to mimic the uneven genomic coverage observed in real data. The dispersion ϕ_*k*_ was sampled from an inverse chi-squared distribution, as described above. Note that, for any given bin *k*, the same sampled values of μ_*k*_ and ϕ_*k*_ were used in all libraries to avoid introducing any DB in the background regions. Finally, reads were evenly distributed between strands and uniformly distributed within each bin.

Each method was run on the simulated data to detect putative DB regions at a nominal FDR of 0.05. Performance was assessed in terms of the observed FDR and detection power. The observed FDR was computed as the proportion of detected regions that did not overlap a known DB site. The detection power was computed as the proportion of known DB sites that were overlapped by detected regions. An overlap was only considered if it occurred between a detected region and the DB subinterval of a known DB site.

#### Sharp DB events

A TF ChIP-seq experiment was simulated with two replicates in two groups. A total of 20 000 binding sites were generated, spaced 10–20 kbp apart on one chromosome. The number of reads at site *i* in group *g* was sampled from a NB(μ_*ig*_, ϕ_*i*_) distribution. The value of ϕ_*i*_ was sampled as previously described. Reads were randomly distributed between strands. Read positions were sampled from *c*_*i*_ − *fX* or *c*_*i*_ + *fX* for reverse- or forward-stranded reads, where *f* is the average fragment length (100 bp) and *c*_*i*_ is the location of the binding site. This recapitulates the strand bimodality of TF binding sites.

For non-DB sites, μ_*ig*_ = 30 for all *g*. To introduce DB, μ_*i*1_ = 45 and μ_*i*2_ = 15 for 500 sites and μ_*i*2_ = 45 and μ_*i*1_ = 15 for another 500 sites. This produces a simple DB event involving a change in the intensity of the entire site. Equal numbers of DB events were added in both groups to avoid any composition bias. Reads for background regions were also added across the genome, as described for the histone mark simulation.

Each DB detection method was applied to the simulated data, to define a set of DB regions at a nominal FDR threshold. The observed FDR and detection power was computed as previously described. This was repeated using thresholds ranging from 0.01 to 0.2. For PePr, only thresholds up to 0.05 were tested.

#### Sharp DB events and fixed dispersions

To evaluate the impact of uncertainty in the dispersion estimates, the TF simulation was repeated without variability in the dispersions across sites. ϕ_*i*_ and ϕ_*k*_ were set to a constant value of 0.05 for all sites and bins, respectively. For non-DB sites, μ_*ig*_ was set to 10 for all *g*. For the DB sites, μ_*i*1_ = 20 and μ_*i*2_ = 0 for 500 sites and μ_*i*2_ = 0 and μ_*i*1_ = 20 for another 500 sites. This creates a realistic scenario with many low-intensity binding events, a small proportion of which are genuinely DB. Methods were applied for DB detection and the observed FDR was calculated at a nominal FDR threshold of 0.05.

### Peak calling software

Peak calling was performed using MACS v2.1 (http://liulab.dfci.harvard.edu/MACS) and the HOMER suite v4.7 (http://homer.salk.edu/homer). MACS was used without model building or duplicate removal. The extension length was set to 100 bp and the genome size was set to the sum of chromosome lengths. When using HOMER, tag directories were constructed from the libraries using the makeTagDirectory command, without removal of non-uniquely aligned reads. The findPeaks command was run on each directory with the style set to ‘histone’ (for histone mark simulations) or ‘factor’ (for TF simulations), a fragment length of 100 bp, the genome size set to the sum of chromosome lengths and no limit on the tags per bp. For both MACS and HOMER, default significance thresholds were used for peak calling.

### Differential binding software

#### Diffbind

For each peak calling method, peak sets for all libraries were loaded into DiffBind v1.14.2 (http://www.bioconductor.org). Peaks were consolidated into a consensus set using a minOverlap of 2, i.e. the peak must be present in at least two libraries. This is the default setting for minOverlap, and seems appropriate for our simulations with two replicates in each group. The setting implies that peaks present in only one replicate will be considered unreliable and will be excluded from the consensus set. Reads were counted into the peak intervals using a fragment length of 100 bp. No removal of duplicates was performed. A contrast was set up between the two groups, with the minMembers parameter set to 2. Finally, detection of DB peaks was performed using the generalized linear model (GLM) methods in edgeR.

#### DBChIP

DBChIP v1.12.0 (http://www.bioconductor.org) was applied in conjunction with MACS for TF simulations. The set of peaks called by MACS in one replicate of each group was used as the representative set for that group. The location of the summit of each peak was supplied to DBChIP, where the negative log-*P*-value computed by MACS was used as the weight of that peak. A consensus set was obtained by merging the peak sets for the two groups. Reads were counted into the consensus peaks for each library, using a fragment length of 100 bp. Each peak was then tested for DB between groups. DBChIP was not used with HOMER because HOMER does not provide summit locations.

#### PePr

PePr v1.0.8 (https://github.com/shawnzhangyx/PePr) was run in differential binding mode with the peak type set to ‘broad’ (for histone mark simulations) or ‘sharp’ (for TF simulations), and the shift size set to half the fragment size, i.e. 50 bp. Artifacts were not removed as duplicates were not simulated. Default values were used for all other parameters. Putative DB sites were identified from each of the up/down output files as those regions with a reported FDR below the nominal threshold.

#### csaw

Analyses used csaw v1.2.1. Reads were extended to 100 bp and counted into windows for each library. The window size was set to 150 bp for simulated histone mark data or 10 bp for simulated TF data. Start positions of adjacent windows were separated by 50 bp along the genome. For filtering, reads were also counted into 2 kbp bins, and the median average abundance of all bins was used as a global estimate of the background abundance. This estimate was downscaled for comparison to the window abundances, based on the difference in the size of the bins and windows. Windows were filtered to retain only those with a two-fold or greater increase in the average abundance above the scaled background estimate. This corresponds to genomic regions where there is substantial enrichment over the non-specific background.

Counts from the remaining windows were tested for significant DB using edgeR v3.10.0. Briefly, an abundance-dependent trend in the NB dispersions was fitted to all windows, using the estimateDisp function. A GLM was fitted to the counts for each window using the trended NB dispersion. The quasi-likelihood (QL) dispersion was estimated from the GLM deviance. An abundance-dependent trend was robustly fitted to the QL dispersions across all windows, and the QL dispersion estimate for each window was shrunk to this trend. Finally, a *P*-value for DB in each window was computed using the QL F-test.

Windows were clustered into genomic regions using a nearest-neighbor approach, where adjacent windows no more than 100 bp apart were placed into the same cluster. A maximum cluster width of 5 kbp was set to avoid chaining. The *P*-values for all windows in each cluster were combined using Simes’ method, and the Benjamini–Hochberg (BH) method was applied on the combined *P*-values from all clusters.

Analyses were conducted using R 3.2.0 and Bioconductor 3.1 for UNIX.

### Case study with embryonic stem cells

Libraries were obtained from the NCBI Gene Expression Omnibus using the accession number GSE53490 (19). Two replicate libraries were present to examine H3K4me3 marking in each of four biological conditions—treated wild-type, treated knockout, untreated wild-type and untreated knockout. This corresponds to Sequence Read Accession files SRR1055323 to SRR1055330, which were converted to FASTQ with the fastq-dump utility from the SRA toolkit. Reads were aligned to the mm10 build of the mouse genome, using Subread v1.4.6 (20) in paired-end mode. Unique mapping was turned on, and any ties were broken with the Hamming distance. BAM files were sorted and indexed using SAMtools v0.1.19 (http://samtools.sourceforge.net). DB detection methods were then applied to identify changes in marking between conditions.

To detect DB events with DiffBind, peaks were called in each library using MACS or HOMER in histone mode. In both cases, the same parameters were used as described for the histone mark simulations, though the fragment length was set to 200 bp based on the insert sizes of proper read pairs in each library (see Supplementary Figure S1). A consensus peak set was constructed using DiffBind, as previously described. Counting was performed for read pairs through the summarizeOverlaps function, without any removal of duplicates. Parallelization was also turned off to simplify processing. Contrasts between groups were set up with minMembers of 2 and the statistical analysis was performed with edgeR.

For csaw, properly paired reads were identified as inward-facing intra-chromosomal pairs that were no more than 600 bp apart. The interval spanned by each proper pair represents the fragment from which the reads were sequenced. The number of fragments overlapping each 150 bp window was counted for each library. Again, the starts of adjacent windows were separated by 50 bp. Background-based filtering of windows was performed as described above. Windows in unassigned contigs or the mitochrondrial genome were also discarded. To remove composition biases, fragments were counted into 10 kbp bins. Normalization factors were computed from these counts, using the trimmed mean of *M*-values (TMM) method without precision weighting. These factors were used to scale the library sizes when testing for DB windows in edgeR's QL framework. Clustering of windows into genomic regions was performed, *P*-values were combined for each region and the BH method was applied as previously described. This analysis was repeated with 1500 bp windows for a low-resolution analysis, where the starts of adjacent windows were separated by 500 bp.

## RESULTS

### Overview of the csaw analysis pipeline

#### Counting reads into windows

csaw takes a set of sorted and indexed BAM files as input, where each file contains aligned reads for one ChIP-seq sample. Either single- or paired-end sequencing data can be accommodated.

To avoid any assumptions about the shape or scope of the DB events, csaw implements a window-based approach to quantify read coverage across the genome. The number of DNA fragments overlapping a genomic window is counted. The window is then shifted by a constant spacing interval and counting is repeated. This is performed for each library in the dataset such that a count is obtained for each window in each library. For single-end data, fragments are imputed by directionally extending each read to the average fragment length (21). The average fragment length can be estimated using cross-correlation plots (22) or can be manually specified. Read extension means that the count for a window is not limited to those reads that directly overlap the window. Even if a read does not overlap the window, its extended version may do so and be counted, provided that the read is adjacent to and facing that window. In this manner, the directionalities of the surrounding reads are implicitly incorporated into the final count for each window. For paired-end data, each fragment is represented by the interval spanned by two reads in a proper pair. This refers to inward-facing reads that are on the same chromosome and separated by a distance less than some maximum threshold, e.g. 600 bp.

The window width is a critical parameter that controls the compromise between spatial resolution and count size. Small windows should be used for sharp enrichment where spatial resolution is critical, e.g. for TFs and histone marks with punctate profiles. For diffuse marks, wider windows will increase the count size and improve DB detection. The choice of window size can be guided by treating the window as the region of contact between the target protein and the genome. For TFs, the contact region may be no more than 10–50 bp. For histones, the width should be at least the length of DNA protected by a nucleosome (about 150 bp). Windows can be as large as several megabases in extreme cases, e.g. when analyzing ChIP-seq data for lamin–DNA interactions. If the choice of window size is not obvious, csaw can provide some assistance. For sharp events, csaw can aggregate the coverage profile across all local maxima in the genome. The window size can then be defined from the width of the peak in the profile. For more diffuse binding, there may not be a single optimal value for the window size. Instead, analyses can be repeated with multiple sizes and consolidated later, to provide comprehensive detection at a range of resolutions.

Note that small window sizes may still be useful for diffuse enrichment. Figure 1 shows a schematic of a diffuse binding event where one subinterval is unbound in the second treatment group. For such complex DB events, small windows can be tiled across the region such that changes in binding within any subinterval of that region will be detected in the corresponding window.

#### Normalization of library-specific biases

csaw automatically adjusts for variable sequencing depth between libraries. In addition, it provides a range of scaling and non-linear normalization techniques to adjust for other biases. To remove composition biases between libraries, reads are counted into 10 kbp bins over the genome for each library. The TMM method (23) is applied to the counts to obtain a set of normalization factors. Alternatively, TMM normalization can be performed directly on the counts for high-abundance windows, i.e. putative enriched regions. This aims to eliminate biases introduced by variable IP efficiencies between libraries. The best choice of normalization strategy depends on the biological problem. TMM normalization with binned counts is more appropriate when global changes in binding are expected. Otherwise, TMM on high-abundance windows may be preferable.

TMM is a scaling normalization method that changes the effective libraries sizes linearly for all genomic regions. A non-linear loess-based normalization method is also implemented to remove trended differences in window counts with respect to abundance, analogous to cyclic loess normalization for microarrays (24,25). More details on the different normalization options are provided in the Supplementary Materials.

#### Assessing DB windows

csaw uses the quasi-likelihood functionality (9,18) of the edgeR package (10) to assess DB between treatment conditions for each window. The QL approach models small integer counts appropriately using negative binomial distributions. This provides accurate, if slightly conservative, type I error rate control while borrowing information between windows to improve statistical power. In particular, it leverages the empirical Bayes functionality of the limma package (26) to model biological variability robustly in the presence of a limited number of replicates. The edgeR package also provides support for arbitrarily complex experimental designs by fitting generalized linear models (8).

Windows with zero or very low counts across all libraries are filtered from the analysis prior to the DB analysis. Their removal reduces computational work, improves dispersion estimation and alleviates the severity of subsequent multiple testing adjustments. The csaw User's Guide discusses a number of different strategies to determine which windows should be filtered. One simple approach is to filter on average abundance as computed by the aveLogCPM function of the edgeR package. In all cases, filtering is done independently of DB testing to avoid loss of type I error control.

#### Aggregating windows into DB regions

From a scientific perspective, it is more meaningful to interpret DB in terms of genomic regions rather than in terms of individual windows. csaw uses a nearest-neighbor clustering method to aggregate adjacent windows into regions. Briefly, a window is placed into a cluster if the gap between the window and the boundaries of the cluster is less than some tolerance. This is repeated until there are no more adjacent windows or the cluster reaches a pre-set maximum allowed width (to protect against excessively large clusters due to chaining effects). The value of the tolerance represents the maximum distance at which two windows are considered to represent the same underlying binding event.

Correct FDR control across regions (clusters of windows) requires special care (12). For each region, the *P*-values of the constituent windows are combined into a single value using Simes’ method. This combined *P*-value represents the evidence against the global null hypothesis for each region, i.e., that none of the windows in the region are DB. The BH algorithm is then applied to the combined *P*-values to control the FDR for detected regions. The same strategy can be used to combine DB results over regions of interest like promoters or gene bodies, for comparison to differential expression analyses. Similarly, results can be consolidated from analyses using different window sizes, for DB detection at a range of spatial resolutions. The window with the most significant DB within each cluster can also be easily identified for purposes of validation or motif discovery.

#### Implementation details

csaw is implemented as an R package. The code is primarily written in R, with C^++^ extensions for greater speed. csaw provides a modular user interface that facilitates a flexible analysis pipeline. It makes extensive use of core Bioconductor packages such as GenomicRanges and Rsamtools (16) to handle genomic data, as well as edgeR and limma for statistical methods. It comes with extensive documentation including a 65 page user's guide. A typical DB analysis with csaw can be fully executed within the R/Bioconductor framework without the need for any additional software, which simplifies installation and improves ease of use.

### Performance for complex DB events

One major aim of the csaw package is to detect complex changes in diffuse binding events between treatment conditions. Such complex DB events might be observed in histone mark datasets. We designed a simulation involving these complex changes, for use in evaluating the performance of different DB detection methods. Specifically, data was simulated for two groups of two replicates where the shape of the binding profile at a DB site changed between groups (Figure 1). (See the ‘Materials and Methods’ section ‘Complex DB events’ for details of the simulation.) We compared the performance of csaw to that of DiffBind in conjunction with two different peak callers. DBChIP was not used here as it is explicitly designed for TF ChIP-seq data. Of the window-based methods, we have previously tested the performance of USeq and diffReps (12). Here, we focus on the newer PePr method.

**Figure 2.**
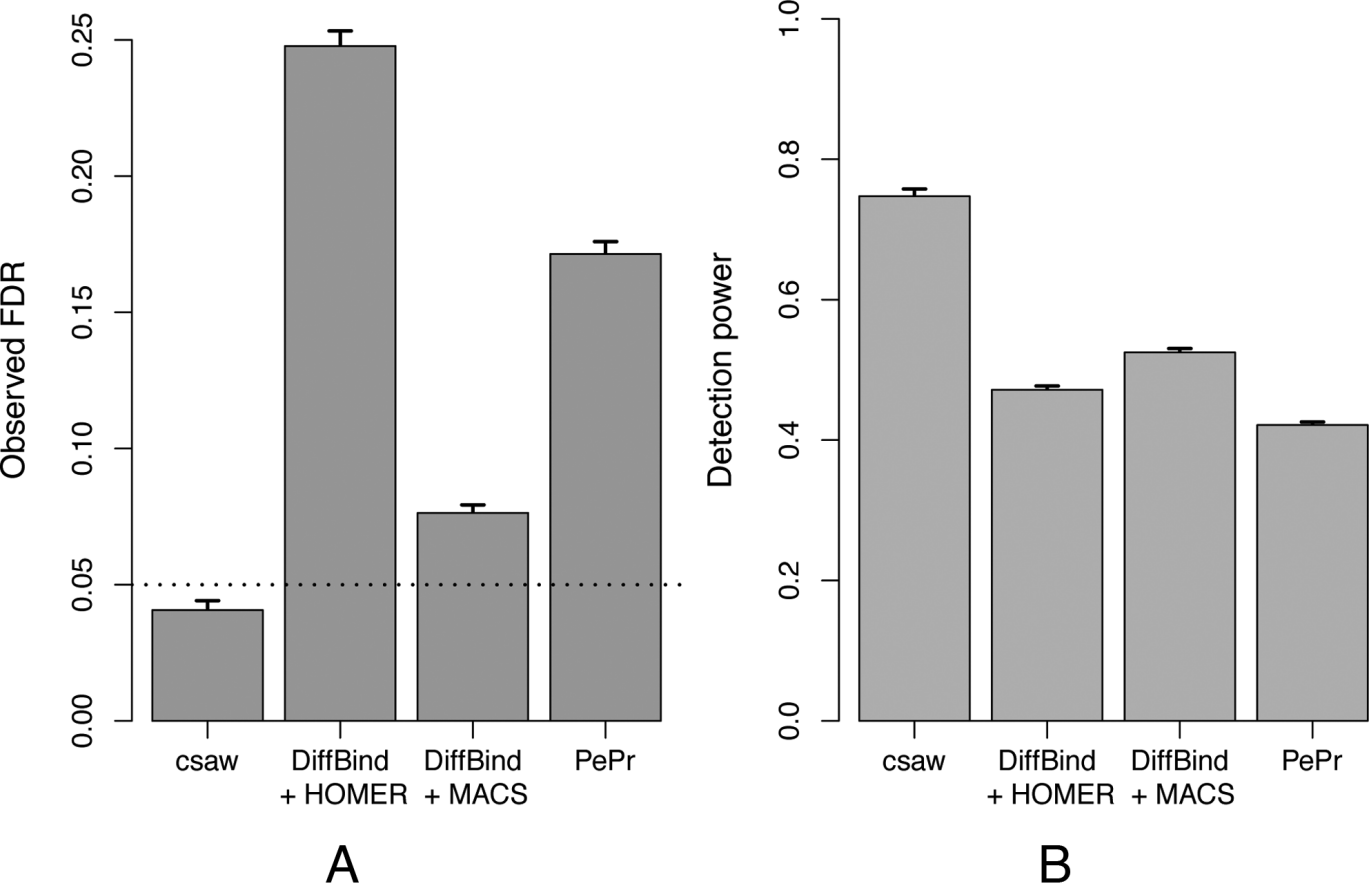
Performance of csaw and peak-based methods for complex DB events in terms of the (**A**) observed FDR and (**B**) detection power. The dotted line shows the nominal FDR threshold, which was set to 0.05 in all analyses. Bar heights and error bars represent the means and standard errors over 10 simulated datasets, respectively.

For all methods, performance was assessed based on the observed FDR and detection power at a nominal FDR threshold of 0.05. The csaw analysis was able to control the FDR below the nominal threshold (Figure 2A). This behavior was robust to changes in the window size, which was initially set at 150 bp. Repeating the analyses with window sizes of 50 and 250 bp yielded an observed FDR of 0.037 and 0.038, respectively, and a detection power of 0.66 and 0.73, respectively. In contrast, the DiffBind-based analyses were liberal, especially so for the DiffBind-HOMER combination. Similar liberalness was observed with PePr. Despite being the only conservative method, csaw was still able to provide a substantial increase in detection power over all other methods (Figure 2B).

### Performance for sharp DB events

**Figure 3.**
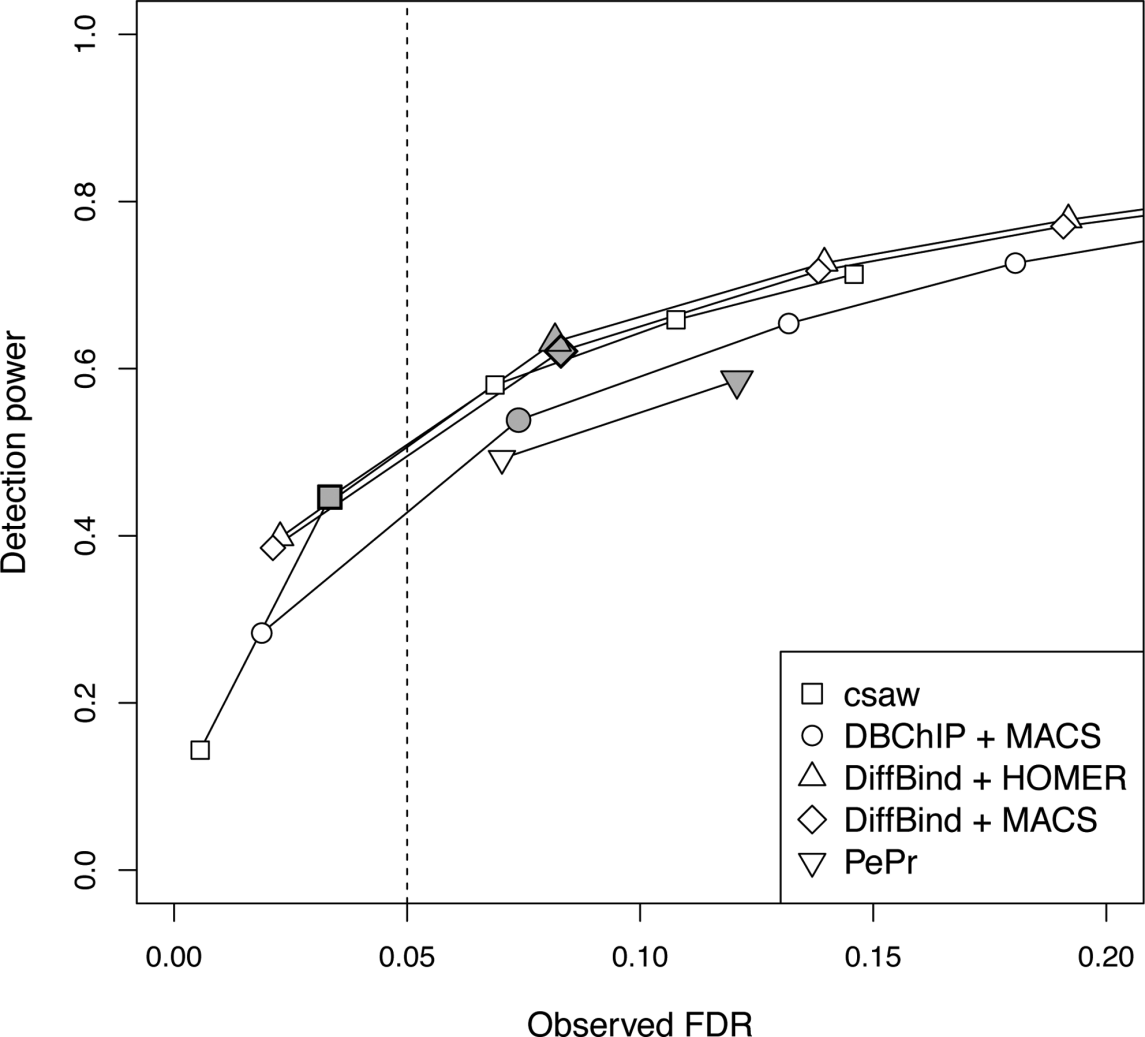
Performance of the different methods when detecting DB for sharp TF peaks. Each curve represents the change in the observed FDR and detection power as the nominal FDR threshold is varied for each method. From left to right, the points on each curve correspond to thresholds of 0.01, 0.05, 0.1, 0.15 and 0.2. Only thresholds of 0.01 and 0.05 are shown for PePr. Shaded points mark the performance of each method at a threshold of 0.05 (dotted line). All values represent means from 10 simulated datasets.

Further simulations were conducted to evaluate the performance of each method on sharp peaks from TF binding experiments. Data was generated for two groups of two replicates, where DB was present between groups at several sites. This involves ‘simple’ DB that occurs across the entire site, i.e. a change in intensity without a change in the binding profile. (See the ‘Materials and Methods’ section ‘Sharp DB events’ for details of the simulation.) csaw was compared to DiffBind-MACS, DiffBind-HOMER and PePr as before. It was also compared to DBChIP (11), which is specifically designed to detect sharp peaks. The performance of each method at nominal FDR thresholds ranging from 0.01 to 0.2 were tested. For PePr, only thresholds up to 0.05 were tested. This is because sites with higher FDR values are not reported, and no options are available to directly specify the nominal FDR.

csaw and DiffBind provided similar detection power at any given value for the observed FDR (Figure 3), whereas slightly less power was observed for DBChIP and PePr. However, only csaw was able to control the FDR below the nominal threshold. For example, at a nominal threshold of 0.05, csaw was the only method to yield an observed FDR that is lower than this threshold (see shading in Figure 3). All other methods are liberal as they yield observed FDRs above this threshold. The same effect can be observed at other thresholds, where the corresponding point on the curve for csaw lies to the left of each threshold while the points for the other methods lie on the right. In fact, at the highest threshold of 0.2 (i.e. rightmost point on each curve except for PePr), only csaw has an observed FDR within the range of the plot. This highlights the liberalness of the peak-based methods in this simulation.

**Figure 4.**
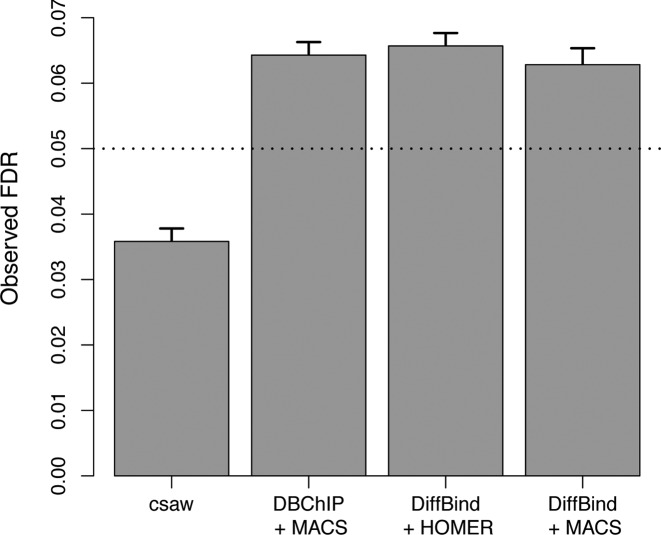
Observed FDR of csaw and the peak-based methods for simulated TF data with constant dispersion. Bar heights represent the mean of 10 simulated datasets, while error bars represent standard errors. The nominal FDR is set to 0.05 and is shown as the dotted line.

DiffBind and DBChIP use edgeR to perform likelihood ratio tests (LRTs) or exact tests to detect DB. In comparison, the csaw package uses the more conservative quasi-likelihood F-test. Unlike the LRT or exact test, the F-test accounts for the uncertainty of estimation of the NB dispersion parameters. We wondered to what extent the liberalness of DiffBind and DBChIP in the above simulation is due to the choice of statistical test. To answer this question, we performed another TF simulation where the NB dispersion parameters were fixed for all sites. (See the ‘Materials and Methods’ section ‘Sharp DB events with fixed dispersion’ for details of the simulation.) Analyses with each method were performed as described for the previous TF simulation, though estimation of tagwise dispersions was turned off in DiffBind. Both DiffBind and DBChIP will use a global common dispersion parameter across all peaks, which should be precisely estimated with negligible error. This should negate any advantage of the F-test over the LRT or exact test. However, even in this favorable scenario, FDR control was still lost by both DiffBind or DBChIP (Figure 4).

### Differential H3K4me3 marking in embryonic stem cells

The utility of detecting complex DB events can be highlighted with some real ChIP-seq data. The chosen study investigates H3K4me3 marking in mouse embryonic stem cells (19). The study contains four groups involving wild-type and *Cfp1* knock-out mice, where marking in each genotype has been assayed before and after treatment with doxorubicin. Each group also contains two biological replicates.

**Figure 5.**
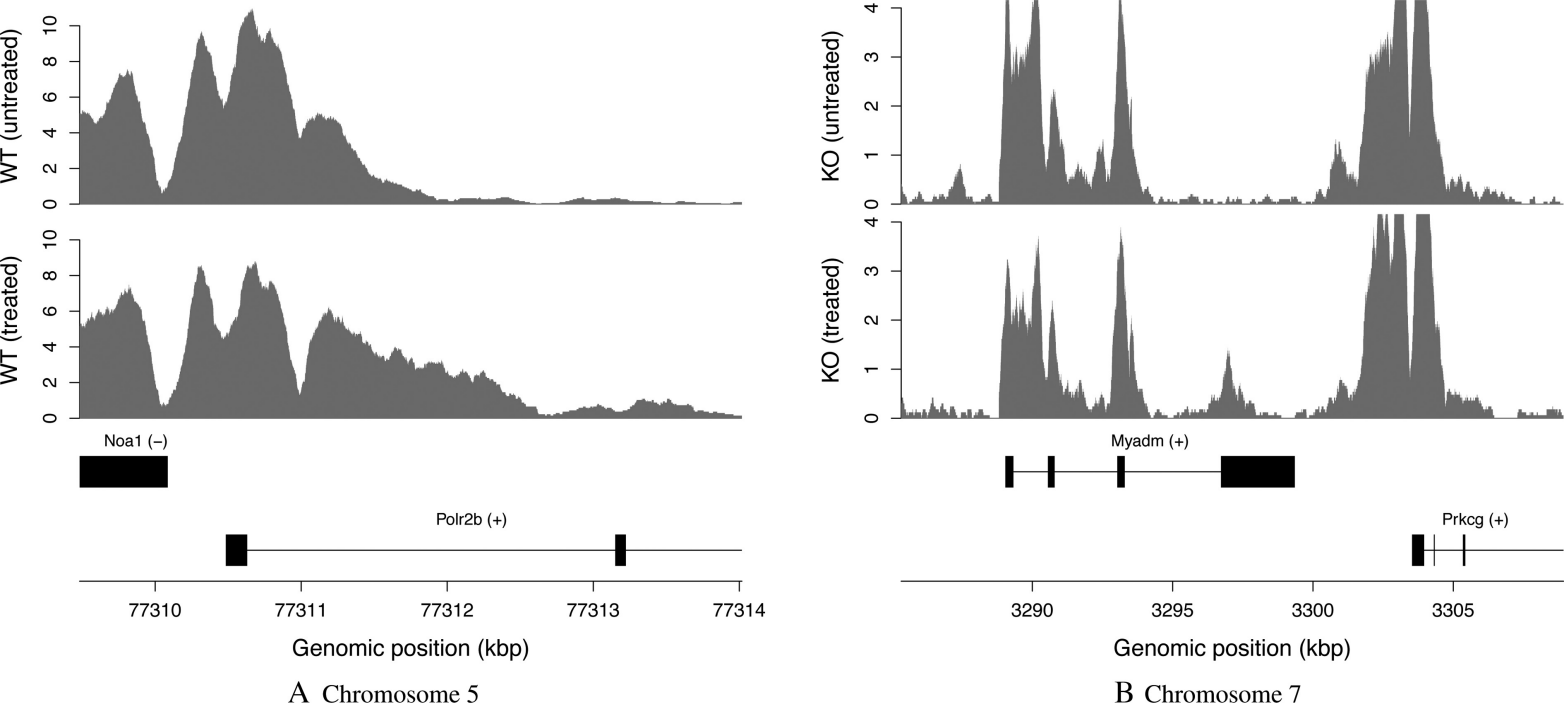
Examples of complex DB events in the H3K4me3 dataset, detected by csaw but not DiffBind at a FDR threshold of 0.05. Each track represents the coverage profile for a single representative library of a group. The profile itself is defined as the number of fragments overlapping each base, transformed into a count-per-million value based on library size. Any genes in the interval are also shown, based on NCBI mouse build 38 annotation. (**A**) DB before and after treatment for wild-type (WT) mice, detected with a FDR of 6.7 × 10^−9^. (**B**) DB before and after treatment for knockout (KO) mice, detected with a FDR of 1.1 × 10^−5^.

The aim of the analysis is to identify changes in marking upon doxorubicin treatment in each genotype. This was done by applying csaw, DiffBind-HOMER and DiffBind-MACS. For csaw, normalization factor estimates are visualized with MA plots in Supplementary Figure S2, while plots of the NB and QL dispersion estimates are shown in Supplementary Figure S3. DBChIP was not used here, as it is limited to analyses of TF ChIP-seq data. PePr was not used for simplicity, given that its window-based analysis is redundant with (and, in the simulations, outperformed by) csaw. For all tested methods, DB comparisons were performed between the treated and untreated groups for each genotype. Putative DB regions were detected at a nominal FDR of 0.05. Coordinates of detected regions were compared between csaw (using 150 bp windows) and DiffBind (using HOMER or MACS) to identify DB regions unique to each method.

csaw detected a number of complex DB events in the H3K4me3 dataset. In Figure 5A, marking spreads throughout the *Polr2b* gene upon doxorubicin treatment. This is potentially interesting as H3K4me3 is a mark of transcriptional activation. Increased marking may correspond to an increase in expression for this gene. However, using DiffBind with MACS or HOMER fails to detect this event. This is because the entire enriched region is defined as a peak, such that the relevant DB subinterval cannot be resolved. Similarly, in Figure 5B, treatment results in an increase in marking in the final exon of the *Myadm* gene. This event is not detected by DiffBind, as peak calling of the low-abundance DB site is confounded by the neighboring high-abundance sites.

The modularity of csaw means that the GLM machinery in edgeR is fully accessible. This allows the specification of more complex comparisons. For example, users can specify an ANOVA-like contrast to identify DB regions with any changes in binding across all groups. One such region is shown in Supplementary Figure S4a, where marking drops upon both treatment and upon knocking out *Cfp1*. Alternatively, users might be interested in regions where the effect of treatment in the wild-type genotype differs from that in the knockout. An example is provided in Supplementary Figure S4b, where marking increases slightly upon treatment in the wild-type but decreases considerably upon treatment in the knockout. Most other pipelines cannot perform these contrasts as they are limited to simple comparisons between two groups.

DiffBind with MACS or HOMER also detects a number of putative DB features that are not found by csaw. Many of these are diffuse regions with weak but consistent DB (Supplementary Figure S5). Peak-based methods provide greater detection power for such regions, as large peaks can collect more read counts than small windows (12). Each subinterval of the region has the same DB status, so no benefit is gained from considering each subinterval separately with small windows. That said, if such events are of interest, they can be identified by simply increasing the window size in csaw. This sacrifices spatial resolution for count size, decreasing power for sharp DB in favor of diffuse DB. Indeed, both examples in Supplementary Figure S5 are detected by csaw when a window size of 1.5 kbp is used. Comprehensive DB detection at a range of resolutions can be achieved by repeating the analysis with different window sizes.

## DISCUSSION

In the simulations, csaw was the only method that was able to control the FDR correctly at or below the nominal level for detected DB regions. This is partially due to csaw's use of the QL F-test, which accounts for uncertainty in the dispersion estimates (9). Indeed, csaw has a modular design that allows the latest functionality in edgeR (or other packages) to be easily implemented in a DB analysis pipeline. In contrast, DiffBind and DBChIP provide wrapper functions around edgeR that limit access to the underlying statistical methods.

However, the use of different tests does not fully explain the liberalness of the peak-based methods. Loss of control is still observed in a special simulation where the tests used by DiffBind and DBChIP should have optimal performance. This is due to the lack of independence between peak calling and DB detection (12). DBChIP selects peaks that are present in either group. This is likely to enrich for regions with spurious DB, as the sites that minimally satisfy this criterion will necessarily be present in one group and not the other. The situation is more subtle with DiffBind, which selects peaks that are present in at least two libraries by default. Sites that minimally satisfy this criterion are those with high-abundance peaks in exactly two libraries. Again, this enriches for sites with spurious DB, i.e. where both peaks are in the same group. Note that the dispersion estimates will also be inflated by the presence of sites with the two peaks in different groups, but—in this simulation, at least—any resulting conservativeness is more than offset by the liberalness from spurious DB. csaw avoids all of these issues as no peak calling is performed.

Loss of FDR control with PePr is demonstrative of the difference between window-level and region-level error rates. In PePr, significant windows are identified before aggregation into regions for reporting (15). However, controlling the window-level error is not equivalent to controlling its region-level counterpart (12). This results in liberalness when results are interpreted in terms of regions. In csaw, Simes’ method is used in conjunction with the BH method to guarantee control of the region-level FDR.

csaw was more powerful than other methods at detecting complex DB events, especially those involving changes in the width of a binding event. This was shown with simulated data and also demonstrated in the H3K4me3 case study. The relatively poor performance of peak-based methods can be attributed to the fact that each binding site is defined as a single peak by MACS or HOMER. If reads are counted from the entire site, strong changes in one subinterval of the site are likely to be masked by the lack of DB in the rest of the site. In contrast, csaw is able to tile small windows across the binding site. Each window corresponds a different subinterval of the binding site, such that the magnitude of any changes in part of the site can be faithfully captured by a window. This improves detection power for these complex events.

We note that the latest MACS software is capable of identifying subpeaks within a single peak interval. This might be expected to improve spatial resolution for analysis of complex DB events. However, the simulated data in Figure 1 shows no obvious subpeaks within the coverage profile for each condition. Similarly, no subpeaks are apparent across the DB subinterval in Figure 5A and Supplementary Figure S4a. It is also challenging to consolidate multiple adjacent subpeaks for each peak into an unambiguous consensus set across all libraries. Indeed, just like region-level FDR control is preferred to window-level control, one must decide whether the FDR should be controlled across all subpeaks or across all peaks. This is not straightforward if each peak interval is aggressively partitioned into many subpeaks.

csaw did not exhibit any power advantage over DiffBind for the TF simulation. This is because the sharp peaks were easily identified by the peak calling software. Note that we used the updated version of MACS (v2.1), which reports more precise peak boundaries than its predecessor (v1.4). In this case, windowing did not provide any useful gain in spatial resolution. Nevertheless, csaw still returned the same performance curve as the best peak-based methods.

The modularity of the csaw package means that the full GLM machinery in edgeR is accessible to the user. This allows csaw to accommodate other experimental designs and DB contrasts. For example, csaw can be used to perform paired analyses or can test for an interaction effect, as illustrated in Supplementary Figure S4b. csaw can also handle other data types beyond complex DB in H3K4me3 marking. We have previously shown that the same window-based method can detect simple DB events in TF, H3K4me3 and H3ac datasets (12). A recent study has also successfully used csaw to analyze differential H3K27me3 marking (27,28). Further interrogation of this dataset reveals that complex DB is present for broad H3K27me3 marks, and that these events can be detected with csaw (Supplementary Figure S6).

While csaw does not require negative control libraries such as input or IgG controls, they can be accommodated into the DB analysis if deemed necessary. A simple approach is to use the controls to refine the filtering step, whereby a window is only retained if the average abundance across the ChIP libraries is substantially greater than that across the controls. Alternatively, csaw can be used to detect DB between ChIP samples and controls to identify absolute binding sites. More generally, the GLM framework means that csaw can incorporate condition-specific controls into a regular DB analysis in several ways. One approach is to include the controls in the linear model so that the log-fold change between conditions for the ChIP samples is compared to that of the controls. Another approach is to normalize the ChIP samples to condition-specific controls and to pass the adjustments to csaw as offsets for GLM fitting (5,29).

In summary, the csaw package provides a window-based approach for *de novo* detection of DB regions from ChIP-seq data. It does so in a statistically rigorous manner, by providing well-considered methods to normalize library-specific biases, to model biological variability and to control the appropriate FDR. The modularity of the package means that its constituent methods can be applied in other pipelines—for example, Manna *et al*. use csaw's normalization strategy in conjunction with promoter-based counting (30). In general, csaw could be used for a window-based analysis of any sequencing data where differences in genomic coverage are of interest, e.g. differential chromatin accessibility in DNase-seq, differential methylation in MeDIP-seq or discovery of unannotated differentially expressed transcripts in RNA-seq (31).

## AVAILABILITY

csaw is freely available under the GPL-3 license for Windows, MacOS and UNIX as part of the Bioconductor project (http://www.bioconductor.org) (17,32). Information about the lastest official release of the software can be viewed at http://www.bioconductor.org/packages/release/bioc/html/csaw.html.

## SUPPLEMENTARY DATA

Supplementary Data are available at NAR Online.

SUPPLEMENTARY DATA
