# Supplementary material for: csaw: a Bioconductor package for differential binding analysis of ChIP-seq data using sliding windows
Source: Nucleic Acids Res. 2015 Nov 17;44(5):e45. doi: 10.1093/nar/gkv1191 (PMC4797262; doi:10.1093/nar/gkv1191)
Supplement: SUPPLEMENTARY DATA [file supp_44_5_e45__index.html]

csaw: a Bioconductor package for differential binding analysis of ChIP-seq data using sliding windows — SUPPLEMENTARY DATA 

# csaw: a Bioconductor package for differential binding analysis of ChIP-seq data using sliding windows

## SUPPLEMENTARY DATA

- SUPPLEMENTARY DATA
