# Supplementary material for: csaw: a Bioconductor package for differential binding analysis of ChIP-seq data using sliding windows
Source: Nucleic Acids Res. 2015 Nov 17;44(5):e45. doi: 10.1093/nar/gkv1191 (PMC4797262; doi:10.1093/nar/gkv1191)
Supplement: SUPPLEMENTARY DATA [file supp_gkv1191_nar-02867-met-n-2015-File012.pdf]

Supplementary Materials For

csaw: a Bioconductor package for differential binding analysis of ChIP-seq data using sliding windows

by

Aaron T. L. Lun[1,2] and Gordon K. Smyth[1,3]

[1]The Walter and Eliza Hall Institute of Medical Research, 1G Royal Parade, Parkville, VIC 3052, Australia

[2]Department of Medical Biology, The University of Melbourne, Parkville, VIC 3010, Australia

[3]Department of Mathematics and Statistics, The University of Melbourne, Parkville, VIC 3010, Australia

24 September 2015

1 Additional detail on normalization methods

1.1 Library-specific biases in a DB analysis

Composition bias is introduced when unbalanced numbers or sizes of DB regions are present between conditions. Regions containing genuine binding sites will pull down more fragments and use up more sequencing resources. This suppresses the representation of other regions with weaker or fewer binding sites. If the magnitude of suppression is different between libraries, spurious differences will be observed in the read counts of non-DB regions. This will increase the false positive rate for the DB analysis. Detection power may also decrease as the suppression effect can reduce the fold change for genuinely DB regions.

Another bias can be introduced during the immunoprecipitation (IP) step. Efficient IP of the target protein will result in strong peaks in read coverage at the binding sites, whereas inefficient IP will yield weak peaks. If the IP efficiency differs across libraries, an "efficiency bias" will manifest as a systematic difference in the strength of the peaks between libraries. This can result in spurious DB between libraries in different groups. Alternatively, differences in efficiency between biological replicates will inflate the variance estimate and reduce detection power. Neither outcome is ideal in a DB analysis.

1.2 Eliminating biases with scaling normalization methods

Scaling methods can be used to remove these biases prior to a DB analysis in csaw. For composition bias, the TMM procedure [22] can be applied on counts collected across large genomic bins. This assumes that most regions in the genome are non-DB background regions and, thus, should have the same coverage between libraries. Any systematic difference in coverage represents composition bias and must be removed. To remove efficiency bias, the TMM procedure is applied on counts collected across high-abundance windows. These windows represent bound regions in the genome, most of which are assumed to be non-DB. Any systematic difference between libraries must represent efficiency bias and is removed.

Some care is required when choosing which scaling method to use for a particular dataset. In particular, what happens when there is a systematic difference in the read counts across binding sites between two libraries? Applying the TMM method on high-abundance windows will eliminate this systematic difference, while applying TMM on background bins will increase it (by eliminating the suppression of the fold change). The correct approach depends on whether this systematic difference represents genuine DB or changes in IP efficiency. If it is the former, the difference is interesting and should be preserved with background-based TMM. Otherwise, it should be removed with window-based TMM. This choice must be guided by external biological knowledge or experimental information – whether the quantity or activity of the target protein changes between conditions, correlations of DB to changes in gene expression, etc.

In the analysis of the H3K4me3 dataset from Clouaire *et al.* [17], all systematic differences were assumed to represent genuine DB. This is based on the role of *Cfp1* in H3K4me3 deposition, such that knocking it out should result in overall changes in marking. Similarly, doxorubicin treatment results in sudden p53-dependent changes in gene expression. This should be accompanied by systematic changes in the H3K4me3 profile, given that H3K4me3 is a mark of transcriptional activition.

1.3 Dealing with trended biases through non-linear normalization

Complex trended biases can also be observed whereby the magnitude of the difference between libraries changes with the average read abundance across libraries. This is refractory to scaling normalization as the required adjustment will vary with abundance. Instead, csaw provides a method for non-linear normalization which is adapted for low count data. For each sample, counts are log-transformed after addition of a continuity correction of 0.5. A loess curve is fitted to the log-count against the average abundance for all windows. The fitted value for each window in each sample is used as the GLM offset for the corresponding observation in the downstream DB analysis. This procedure is analogous to the fast loess method [24], used for

normalization of normally-distributed microarray intensities. It is equivalent to constructing an "average" library where the count for each window is set at the average count across all libraries, and then normalizing each individual library against this average library.

It is worth elaborating on why this adaptation is necessary for data with low counts. Direct application of the fast loess method requires log-transformation of the counts, such that normalization will remove non-zero log-fold changes between libraries. This means that the A-value (i.e., average log-count) is used as the covariate for loess fitting. The adaptation presented here uses the average abundance (i.e., log-average count) instead. The A-value is not stable for windows with strong DB where the count for one library is close to zero. These windows will have low A-values due to the log-transformation of a near-zero count into a large negative value. As such, the covariate interval across low A-values will be enriched for DB windows. This may violate the assumption of loess-based normalization, i.e., that most windows are not DB throughout the covariate range. The fitted trend and offsets will subsequently be incorrect. This is avoided with the modified procedure, where the improved stability of the average abundance for near-zero counts avoids accumulation of DB windows at low covariate values.

2 Comparisons with other methods

Here we report briefly on other methods that were tested but not reported in the formal comparisons in Sections 4 and 5 of the main article.

SICER [3] is another peak caller aimed specifically at histone modification data. We repeated the histone mark simulations using SICER instead of HOMER to call the peaks. The performance of DiffBind with SICER was similar to that reported for DiffBind with HOMER. SICER was run using a window size of 200 bp, gap size of 400 bp and E-value of 1.

multiGPS [6] is another method to detect sharp DB events between conditions. Unlike the other methods discussed in this article, multiGPS is not easily classified as either a window- or peak-based method. Rather, it assigns each read to a single putative binding site and then tests for differences between conditions using edgeR. We found multiGPS to be slower and more conservative than the methods compared in the main article. In the TF simulation, multiGPS detected 2% and 15% of all DB events at FDR thresholds of 0.01 and 0.2, respectively, compared to 17% and 75% for csaw. This low power appears to be mainly due to conservativeness during the initial detection of binding sites, such that few sites are passed to edgeR for DB testing. For the histone mark simulation, we were unable to obtain results from multiGPS in a reasonable amount of time. This may be because of the number of expectation-maximization iterations required, and because multiGPS is designed to detect sharp binding events.

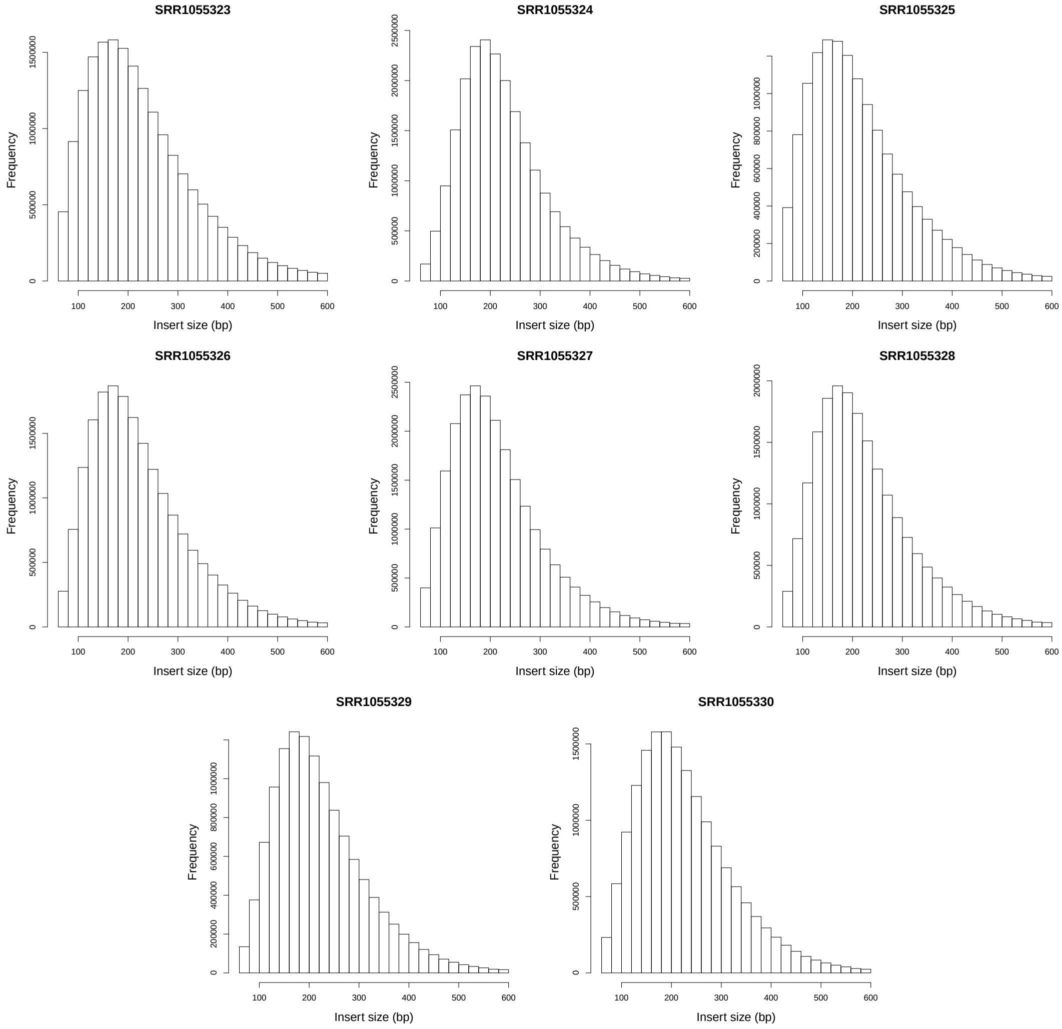

Figure S1: Distribution of insert sizes for properly paired reads in the H3K4me3 data set. Intra-chromosomal read pairs were considered to be proper if they were inward facing and no more than 600 bp apart. Insert sizes were defined as the distance between the $5'$ ends of such paired reads.

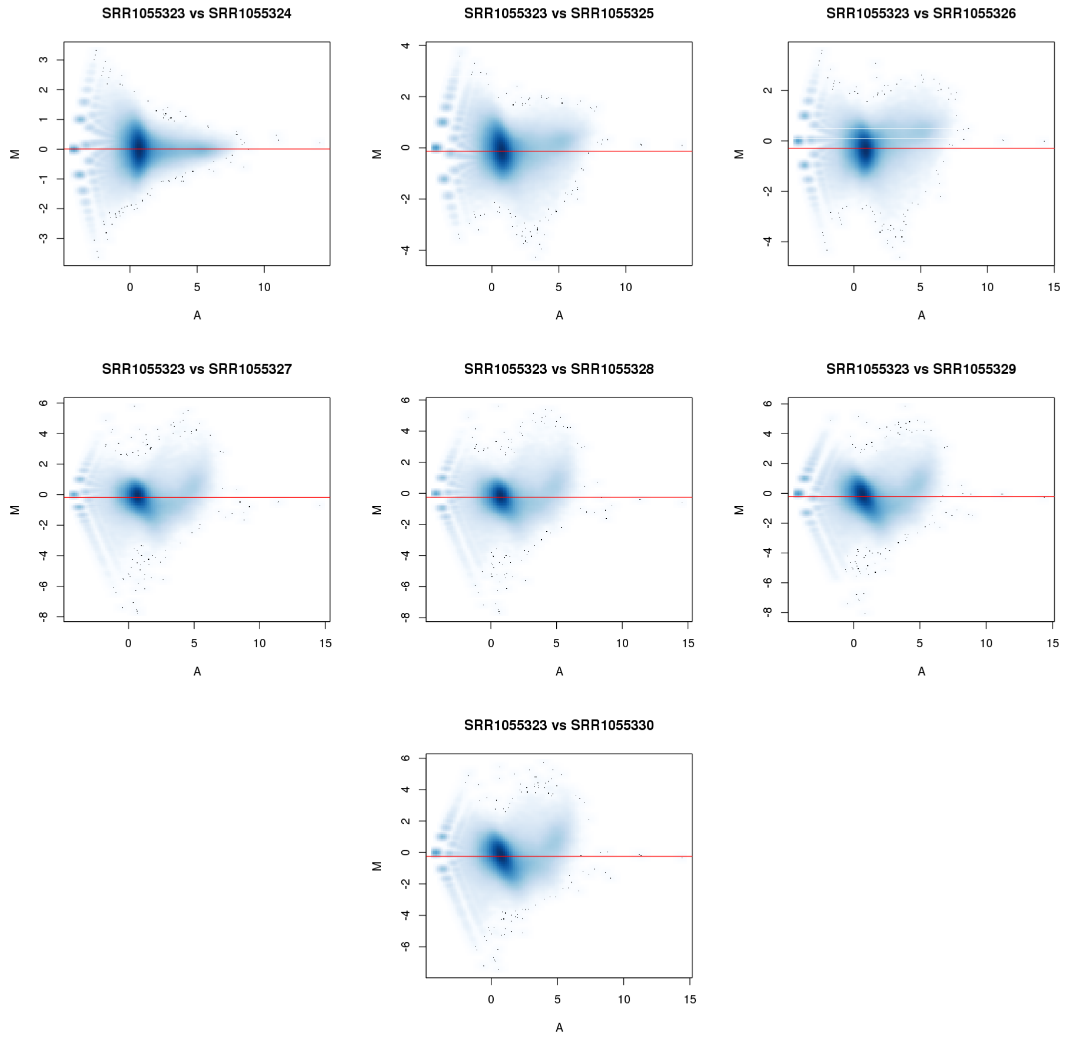

Figure S2: MA plots between pairs of libraries in the H3K4me3 data set, using SRR1055323 (i.e., the first replicate of the wild-type untreated group) as the reference library. M- and A-values were computed as the difference between and average across libraries, respectively, of the $\log_2$-count-per-million values for 10 kbp bins. The depth of colour is proportional to the density of points in each plot. The red line corresponds to the log-ratio of the normalization factors for the two libraries in each plot.

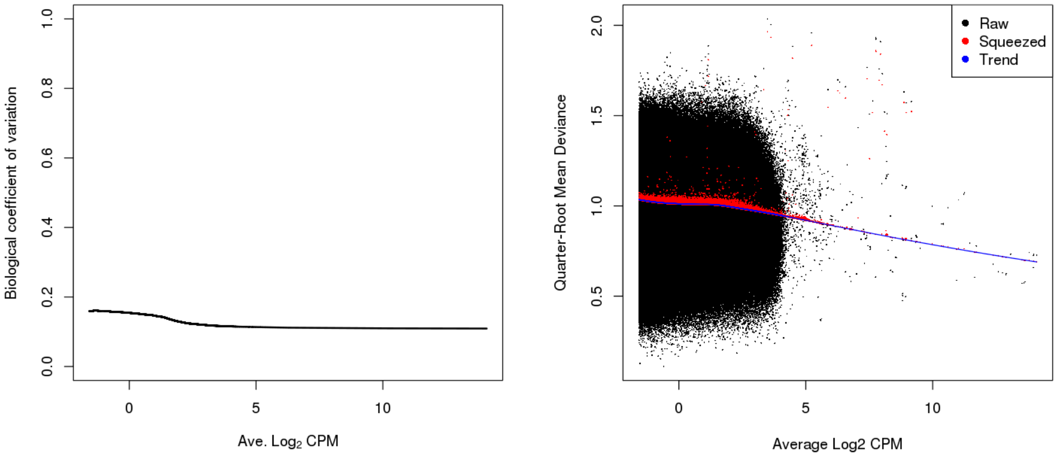

Figure S3: Estimates of the trended NB (left) and QL dispersions (right) for 150 bp windows in the H3K4me3 data set. The biological coefficient of variation is the square-root of the NB dispersion. QL dispersions are shown as quarter-root values to improve resolution around unity. QL estimates are shown before (black) and after (red) shrinkage towards an abundance-dependent trend (blue).

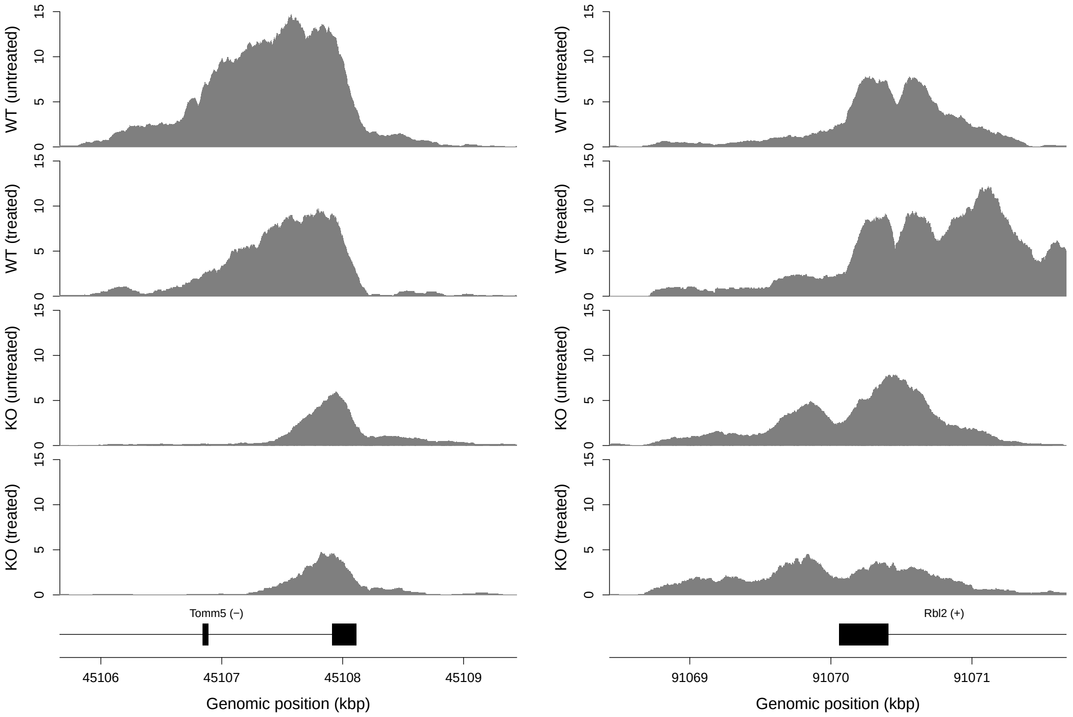

(a) Chromosome 4 **(b)** Chromosome 8

Figure S4: Examples of DB events in the H3K4me3 data set, detected by csaw with non-trivial contrasts at a FDR threshold of 0.05. Each track represents coverage by fragments-per-million in a representative library for each group. Annotated gene models are also shown. (a) DB across all four groups in an ANOVA-style comparison, detected at a FDR of 1.9×10^{-66}. (b) DB due to differences in the effect of doxorubicin treatment between genotypes, detected at a FDR of 5.7×10^{-13}.

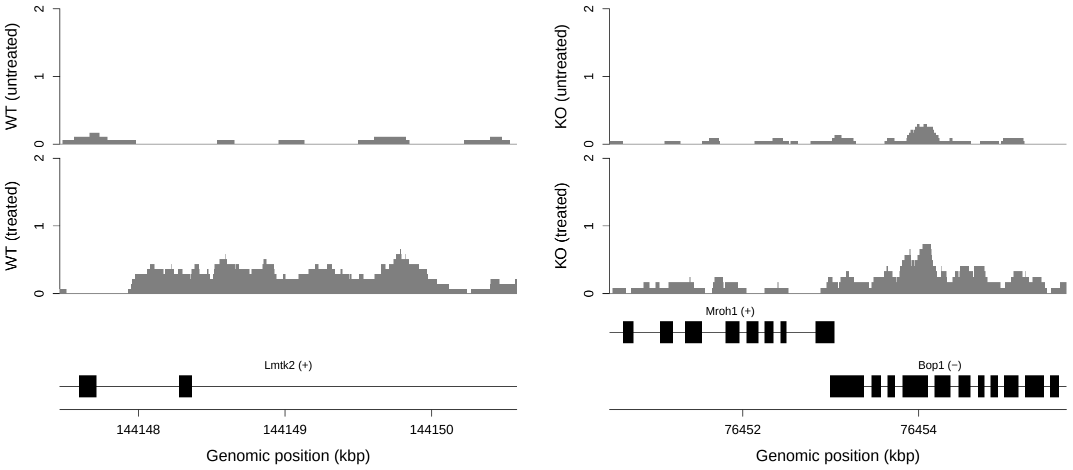

(a) Chromosome 5 **(b)** Chromosome 15

Figure S5: Examples of DB events in the H3K4me3 data set, detected by DiffBind but not csaw at a FDR threshold of 0.05. Each track represents coverage by fragments-per-million in a representative library for each group. Annotated gene models are also shown. (a) DB before and after treatment for wild-type mice, detected at a FDR of 5.7×10^{-15}. (b) DB before and after treatment for knock-out mice, detected at a FDR of 5.6×10^{-13}.

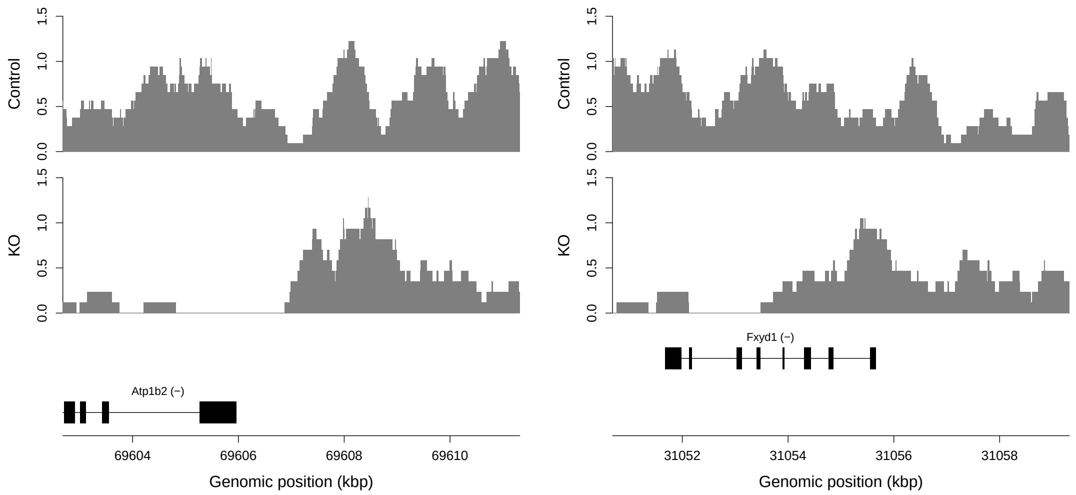

(a) Chromosome 11 **(b)** Chromosome 7

Figure S6: Examples of complex DB events in a H3K27me3 data set, detected by csaw at a FDR threshold of 0.05 as described by Holik *et al.* [28]. This study focuses on marking in mouse lung epithelial cells, before (control) and after knocking out the histone methytransferase *Ezh2* (KO). A DB subinterval between control and KO is shown for each broadly marked region in (a) and (b), detected at FDRs of 3.6×10^{-5} and 4.6×10^{-5} respectively. Each track represents coverage by reads-per-million in a representative library for each group, where coverage is computed using a 500 bp smoothing window to improve visualization of sparse data. Annotated gene models are also shown.
